# HCV-Related Nervous System Disorders

**DOI:** 10.1155/2012/236148

**Published:** 2012-07-30

**Authors:** Salvatore Monaco, Sergio Ferrari, Alberto Gajofatto, Gianluigi Zanusso, Sara Mariotto

**Affiliations:** Section of Neuropathology, Department of Neurological, Neuropsychological, Morphological and Motor Sciences, University of Verona, Verona 37134, Italy

## Abstract

Chronic infection with hepatitis C virus (HCV) is associated with a wide spectrum of extrahepatic manifestations, affecting different organ systems. Neurological complications occur in a large number of patients and range from peripheral neuropathy to cognitive impairment. Pathogenetic mechanisms responsible for nervous system dysfunction are mainly related to the upregulation of the host immune response with production of autoantibodies, immune complexes, and cryoglobulins. Alternative mechanisms include possible extrahepatic replication of HCV in neural tissues and the effects of circulating inflammatory cytokines and chemokines.

## 1. Introduction

Chronic infection with hepatitis C virus (HCV), a hepatotropic and lymphotropic agent, is a growing global health issue affecting an estimated 170 million people [1]. In addition to be a leading cause of chronic hepatitis, cirrhosis, and hepatocellular carcinoma (HCC), chronic HCV infection has been associated with more than 30 extrahepatic manifestations (EHMs), affecting a large proportion of infected patients [2]. Many EHMs, including a number of neurological conditions, are immunologic/rheumatologic in nature, as a consequence of B-cell proliferation with ensuing production of monoclonal and polyclonal autoantibodies displaying rheumatoid factor activity or cryoglobulin properties [3]. In addition, lines of evidence suggest that the brain, but not peripheral nerves or skeletal muscles, is a permissive site for viral replication, as evinced from quasispecies analysis and the detection of replicative intermediate forms of HCV RNA and viral proteins within the central nervous system (CNS) [4]. Additional mechanisms, contributing to neurological dysfunction, are possibly related to the effect of circulating inflammatory cytokines and chemokines reaching brain tissues across altered sites of the blood-brain barrier. Cryoglobulinemia, the most frequent and best-studied EHM of HCV infection, is detected in up to 50% of HCV-infected patients, inducing symptomatic disease in nearly 15% of cases. Cryoglobulins (CGs) are cold-precipitable immunoglobulins, which, following vascular deposition, elicit inflammation and occlusion of small- and medium-size blood vessels. Three types of CG are recognized, either essential or secondary to autoimmune disorders, chronic infections, and lymphoproliferative diseases [5]. Type I CG (10–15% of cases) is monoclonal Ig; type II CG, a mixture of monoclonal Ig rheumatoid factor and polyclonal IgG, accounting for 50–60%, is typically found in patients with chronic HCV infection or primary Sjögren syndrome; type III CG, polyclonal IgG and IgM rheumatoid factor, is seen in lymphoproliferative disorders, chronic infections, and autoimmune diseases [6]. Up to 95% of type II and III CG, or “mixed CG” (MC), are associated with chronic HCV/HIV infection. Mechanisms of CG-induced ischemic tissue damage are secondary to lymphocytic microvasculitis and/or necrotizing arteritis, with transmural fibrinoid necrosis, thrombotic lumen occlusion, and polymorphonuclear cell infiltration. Typical clinical manifestations of symptomatic CG include cutaneous purpura, arthralgias, peripheral neuropathy, and membranous proliferative glomerulonephritis [5, 7]. About 17–60% of patients with CG develop peripheral neuropathy, often at disease onset, while CNS involvement occurs in approximately 6% of cases. In addition to causing vascular damage, CGs represent an independent risk factor for carotid plaque formation, hepatic fibrosis, and liver steatosis. Less frequent EHMs of HCV infection, representing potential threats for neurological involvement, include non-Hodgkin's lymphoma, diabetes, thyroid abnormalities, and rheumatological diseases. Importantly, most of EHMs may improve or even resolve after antiviral treatment, especially in subjects attaining a sustained virological response. The spectrum of CNS and neuromuscular disorders associated with chronic HCV infection is reported in Table 1.

## 2. Neurological Manifestations

 HCV-related CNS complications encompass a wide spectrum of disorders ranging from cerebrovascular events to autoimmune syndromes. However, their relatively low frequency, in addition to the heterogeneity of neurological manifestations, and the paucity of pathological observations, largely preclude the achievement of reliable information as to the pathogenesis of different syndromes. 

Acute cerebrovascular events, including ischemic stroke, transient ischemic attacks, lacunar syndromes, or rarely hemorrhages, have been reported in HCV-infected patients [8–10], being the initial manifestation of HCV infection in some cases [11]. The occurrence of occlusive vasculopathy and vasculitis are well-known events [12, 13]. Isolated CNS vasculitis has been coupled with angiographic evidence of multiple focal narrowing of cerebral arteries, and full recovery has been achieved with corticosteroids and cyclophosphamide [14]. In some patients, CNS ischemic changes may occur in the setting of an antiphospholipid-associated syndrome [15], or in association with antineutrophil cytoplasmic antibodies. Recently, HCV has been connected with the metabolic syndrome and evidence has been provided that HCV infection represents an independent risk factor for increased carotid wall thickness and plaque formation, thus contributing to significant cerebrovascular mortality, especially in patients with elevated HCV-RNA levels [16]. 

Acute or subacute encephalopathic syndromes, clinically characterized by cognitive impairment, confusion, altered consciousness, dysarthria, dysphagia, and incontinence, have been associated with diffuse involvement of the white matter in HCV chronically infected patients with CG and/or circulating anticardiolipin antibodies. An ischemic pathogenesis of these rapidly evolving syndromes is supported by MRI findings showing small lesions in subcortical regions and periventricular white matter. Moreover, severe and diffuse infra- and supratentorial white matter alterations, highly suggestive of vasculitis, are observed in subjects with coincidental systemic vasculitis. Pathological evidence of CNS vasculitis-induced ischemic damage was first provided in a patient with MC, peripheral neuropathy, and relapsing multiinfarct encephalopathy [17]; in this case, neuropathological examination showed multiple ischemic lesions, 0.5–3 mm in diameter, in the white matter of cerebral hemispheres and cerebellum, and parenchymal infiltration and accumulation of lymphocytes around small vessels. The occurrence of possible vasculitis-induced ischemic changes has been also claimed in a patient with chronic HCV infection, MC, Sjögren syndrome, and sensory neuropathy, who developed skin vasculitis and leukoencephalopathy over a three-year period [18]. Taken together, these reports suggest that active HCV infection can be responsible for acute/subacute white-matter involvement, when associated with CG, coagulation disorders, or systemic vasculitis. 

**Figure 1:**
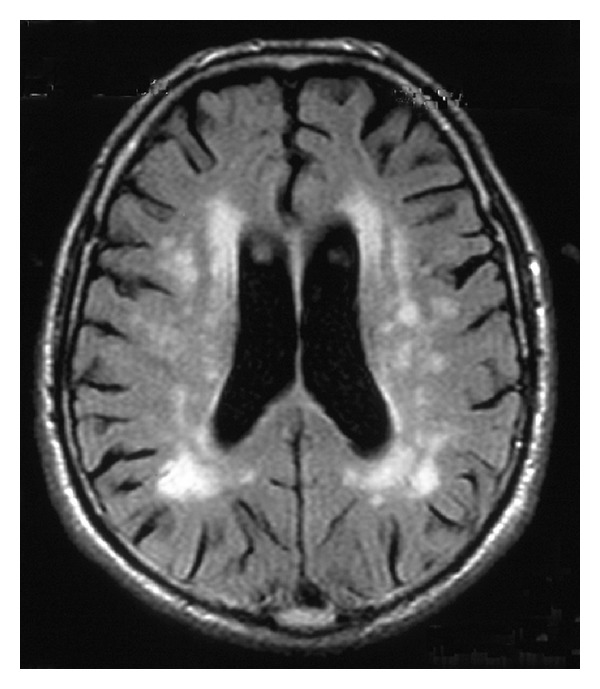
Axial FLAIR MRI of the brain showing periventricular hyperintense areas in a patient with chronic HCV infection and cognitive changes.

In addition to encephalopathic syndromes, slowly evolving cognitive decline, clinically characterized by impairment of attention, executive, visual constructive, and spatial functions, has been correlated to an increased occurrence of periventricular white matter high intensity signals (WMHISs) on T2-weighted MRI [19]. In these patients, a relationship between CG level and number of impaired cognitive functions was observed, while no correlation was found with systemic manifestations of CG, including peripheral neuropathy. WMHIS changes likely reflect the occurrence of small vessel disease, which leads to chronic hypoperfusion of the white matter and local alteration of the blood-brain barrier in anatomical regions where precapillary arterioles are widely spaced and present a poor anastomosing network (Figure 1).

The spectrum of CNS syndromes encountered in HCV patients is not limited to the foregoing vasculitic and vasculopathic forms, but also includes inflammatory disorders, such as acute encephalitis, encephalomyelitis, and meningoradiculitis/polyradiculitis. There are reports of patients with rapidly evolving leukoencephalitis with microglial nodules and perivascular T-cell infiltrates in association with HCV genome presence [20] or fatal progressive encephalomyelitic syndromes, pathologically characterized by neuronal loss and perivascular lymphocyte cuffing in the brainstem and cervical spinal cord [21]. In these cases, available evidence suggests the occurrence of an immune-mediated process induced by HCV, rather than a direct effect of the virus. 

Sacconi et al. described a patient with acute disseminated encephalomyelitis (ADEM), an autoimmune postinfectious CNS disease, developing after HCV infection and responsive to steroid therapy, further supporting the role of cellular immunomediated mechanisms in CNS complications of HCV infection [22]. Chronic HCV infection may also induce humoral-mediated demyelination, sequentially or simultaneously involving the CNS and PNS. Recurrent episodes of CNS and PNS demyelination suggestive of antibody-mediated autoimmunity or, in alternative, of a direct cytopathic effect of the virus, have been reported in a patient with active HCV replication [23]. Relapsing forms of central and peripheral demyelination, worsened by interferon treatment, have also been described [24]. 

**Figure 2:**
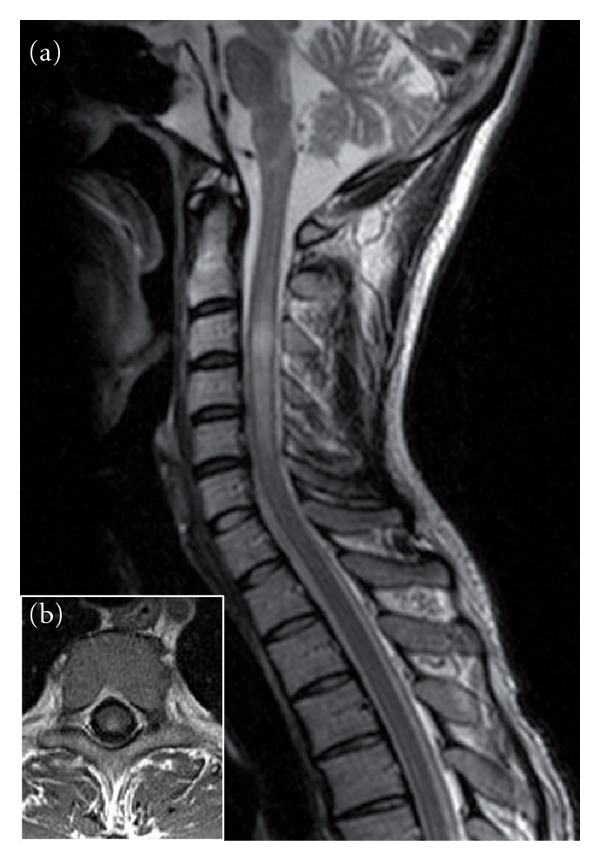
Sagittal (a) and axial (b) T2-weighted MRI sequences disclose cervical spinal cord hyperintensity in a patient with HCV-related myelitis.

Additional examples of HCV-triggered demyelination are observed in patients with myelitis. Myelitis is considered an infrequent neurological complication in patients chronically infected with HCV, although available evidence suggests that a high percentage of patients with recurrent inflammatory transverse myelitis, but not monophasic myelitis, test positive to anti-HCV antibodies and have serum HCV-RNA [25]. HCV-related myelitis occurs acutely [26] or subacutely [27, 28], the neurological presentation ranging from transverse myelitis to acute partial transverse myelopathy, sensory ataxia, or spastic paraplegia; many patients present a recurrent course and have a multisegmental spinal involvement at MRI, usually at cervical and thoracic levels (Figure 2). Notably, patients with negative imaging have been reported. A common feature of HCV-associated myelitis is the presence of circulating anti-HCV antibodies, but not serum CG or HCV-RNA sequences in the CSF. Neuropathological examination of spinal cord has been performed in a few subjects. Biopsy-proven acute demyelination, accompanied by parenchymal and perivascular infiltration of macrophages and lymphocytes, but not vasculitis, has been reported in a 46-year-old man with a history of recurrent myelitis and chronic HCV infection [29]. Biopsy specimens of the spinal cord were negative for HCV antigens and HCV-RNA; on the contrary, anti-HCV antibodies, but not HCV-RNA, were found in the CSF. Necrotic changes in association with proliferation of hyalinized small vessels and infiltration by macrophages and T lymphocytes, mimicking neuropathological features of Sjögren syndrome, have been reported in spinal cord biopsies of a patient with stepwise progressive longitudinal myelopathy and chronic HCV infection, suggesting an ongoing immune-mediated and ischemic pathogenesis [30]. 

## 3. Cognitive/Neuropsychological Symptoms 

More than half of patients with chronic HCV infection complain of “brain fog” (fatigue, impaired concentration, and poor memory) and have a reduced quality of life, regardless of the severity of liver involvement or virus replication rate. Fatigue, cognitive dysfunction, and mood alterations display a profound effect on social and physical functioning, thus further impacting health-related quality of life (HRQL). In earlier studies, fatigue was shown to have a major functional role in patients with chronic HCV infection [31]. Chronic fatigue is perceived as a sensation of physical and mental exhaustion, and, when severe, it is accompanied by deficits of attention tasks, anomia, and word-finding difficulties, in the absence of verbal memory or cognitive ability impairments. In addition, some HCV patients with severe fatigue also complain of muscle and joint pain, sleep disturbances, restless leg syndrome, headache, and depression. Alterations in brain metabolism and neurotransmission, responsible for dysfunction of the ascending reticular activating system, putamen, globus pallidus, and the limbic system, are associated with chronic fatigue. Forton et al. [32] using magnetic resonance spectroscopy (MRS) found elevated choline/creatine (Cho/Cr) ratio in the basal ganglia and frontal white matter of HCV-infected patients, which was not related to the degree of liver disease, viral genotype, or other factors. These authors first suggested that the above changes were secondary to microglial activation, as an effect of HCV brain infection or peripheral cytokines. On the other hand, Weissenborn et al. [33] showed a decrease of the N-acetyl-aspartate(NAA)/Cr ratio in the frontal grey matter of HCV-patients, but no changes of the Cho/Cr ratio. Both findings have been confirmed using a different approach for MRS analysis, suggesting the occurrence of increased cell membrane turnover and decreased neuronal function [34]. More recently, the study of 53 HCV-positive patients with mild liver involvement and neuropsychiatric symptoms, disclosed increased Cho and myo-inositol concentrations in basal ganglia and white matter and increased Cr, NAA, and N-acetyl-aspartyl-glutamate in basal ganglia [35], findings consistent with HCV-induced chronic cellular inflammation. A neurochemical basis for chronic fatigue in HCV-infected patients was suggested by the observation that treatment with ondansetron, a competitive antagonist of serotonin receptors, was effective in ameliorating fatigue in a patient with HCV infection. A significant improvement of the fatigue and depression scores with ondansetron was also found in a placebo-controlled randomized study involving 36 patients with chronic HCV infection [36]. These findings further support a major role for serotoninergic pathway dysfunction in causing fatigue and are in keeping with data showing decreased serum tryptophan levels and related reduction in serotonin synthesis [37, 38]. Alterations in mesencephalic/hypothalamic serotonin (SERT) and striatal dopamine transporter (DAT) binding capacity have been documented by single-photon emission tomography (SPECT) in HCV patients with pathological performance at psychometric tests [39]. More recently, an investigation of 15 HCV patients reporting neuropsychiatric symptoms was performed by combining neuropsychological tests, 18F-fluoro-desoxy-glucose (FDG) positron emission tomography (PET), and SERT; results showed significant reduction in striatal and midbrain dopamine availability and reduced metabolism in limbic, frontal, parietal, and temporal cortices, confirming a major role for defective dopaminergic transmission in causing cognitive impairment in HCV-infected patients [40]. 

Among EHM affecting HRQL, sexual dysfunction and psychiatric disturbances play a major role. Most HCV patients present depression and anxiety, and, using DSM-IV criteria, it has been found that 28% of chronically HCV-infected subjects have depression. In addition, about 15% of patients may suffer of recurrent brief depression [41], a common subtype of cognition-impairing affective disorder, sharing many indicators with major depressive disorder, except the duration of depressive episodes. The occurrence of depression has been attributed to psychological factors, or to specific determinants, including immune mechanisms, derangement of the blood-brain-barrier integrity, viral replication within the CNS, iatrogenic factors, or altered dopaminergic and serotoninergic transmission. Admittedly, mental instability and depression are factors limiting the adherence to specific antiviral treatment, such as conventional interferon formulations or pegylated interferons [42]. While the emergence of mild depression during interferon treatment can be safely managed with antidepressant at low doses, in moderate to severe depression, which affects up to 44% of patients in antiviral therapy, is mandatory to reduce or discontinue interferon treatment, especially in the presence of active suicidal ideation. Proposed neurobiological mechanisms of interferon-induced depression remain to be elucidated, although changes in the hypothalamic-pituitary adrenal axis and in the central catecholamine and serotonin systems, as well as downregulation of serotonin synthesis, have been claimed. However, these mechanisms remain putative and additional studies are warranted [43].

To date, cognitive impairment in chronic HCV infection has been reported in most studies, with the exception of two reports, showing unimpaired cognitive performance despite impaired quality of life in patients enrolled after diagnosis at blood donation [44], and no deficits in attention, adaptive behavior, and intelligence in young HCV-positive children and adolescents with hemophilia [45]. Impaired ability in sustained attention and decreased concentration and psychomotor speed were described earlier [33, 46], although these changes are also found in mildly fatigued patients. Fontana et al. [47] showed prevailing alterations in verbal recall and working memory in about one third of HCV-infected patients, including subjects with liver cirrhosis and substance abuse history. In this study, depression scores were found to be predictors of cognitive impairment. The study on a small cohort of homogeneous state-infected population with mild liver involvement and similar history of iatrogenic HCV exposure reported alterations in general memory, sustained attention, and delayed auditory recognition; importantly, fatigue correlated only with delayed auditory memory recall ability [48]. Investigations employing neurophysiological tests of cognitive processing, such as P300 event-related potentials, have revealed delayed peak latencies and reduced amplitudes in cognitively impaired HCV-infected patients. The use of P300, as an independent measure of cerebral information processing, has the advantage of avoiding the bias of confounding factors such as fatigue or depression, and, in addition, represents a sensitive marker of deranged cortical activation associated with conscious attention. Indeed, investigation of a large population of patients with chronic HCV infection has disclosed the occurrence of subclinical cognitive dysfunction in 18% of subjects; in these cases, delayed peak latencies and reduced amplitudes of P300 largely correlated with fatigue [49]. 

While definitive conclusions regarding the pathogenesis of cognitive dysfunction, fatigue, and depression in HCV infection require further elucidation, many recent data support a major role for the virus itself in causing CNS pathology. Accordingly, the detection of negative-strand HCV RNA in brain tissues of infected patients has suggested that HCV replicates within the CNS; in addition, the diversity of viral quasispecies between the CNS and liver supports an independent life of the virus within the brain [4]. CNS-specific HCV quasispecies have been found to share their genomic sequences with those detected in lymphoid tissues and peripheral blood mononuclear cells (PBMC), but not with liver and serum HCV variants. These molecular data have suggested that infected PBMC could mediate HCV entry in the CNS [50–54] by a “Trojan horse” mechanism [46]. Furthermore, the detection of HCV proteins in macrophages/microglia and astrocytes of postmortem brain samples from patients coinfected with HCV/HIV or monoinfected with HCV suggests a major role for these cells in supporting replication [55]. More recent studies indicate that the brain microvascular endothelial cells (BMECs) are a preferential site of HCV tropism and replication. The demonstration that infection of BMEC causes apoptosis in vitro has suggested that alterations of the blood-brain-barrier could be responsible for microglia activation, following the entry across the brain of inflammatory cytokines and chemokines [56].

## 4. Peripheral Neuropathies 

**Figure 3:**
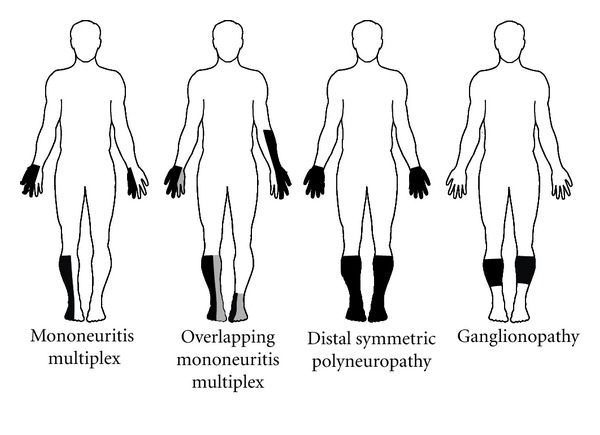
Clinical patterns of PNS involvement in HCV infection.

The PNS is variably affected in HCV-infected patients, mainly depending on the presence and type of CG, associated comorbidities, and iatrogenic factors. In HCV-associated type I CG, the involvement of PNS is rare, and, therefore, the pathogenesis is not entirely understood; our experience is consistent with axonal forms of polyneuropathy, pathologically characterized by perivascular infiltrates, endoneurial purpura, and microangiopathy, overall suggesting an ischemic pathogenesis linked to endoneurial microcirculation obstruction [57]. Conversely, in patients with HCV-associated MC, the involvement of the PNS ranges from 26% to 86%, in accordance with the disease stage and the clinical/electrophysiological protocols for neuropathy ascertainment. In most cases, pathological features are indicative of ischemic nerve changes, as a consequence of small vessel vasculitis, or, less frequently, necrotizing arteritis of medium-sized vessels [58]; the presence of circulating CG is predictive of severe PNS involvement and a cryocrit level higher than 5% is detected in aggressive vasculitic forms, with recurrent purpura. In patients without CG, immune complexes or HCV-induced autoimmune mechanisms may play a pathogenetic role in inducing vascular and perivascular inflammation, which may be driven by an intrinsic nerve population of immunocompetent and potentially phagocytic cells [59]. The possible role of HCV in inducing vascular inflammation is suggested by pathological/molecular studies of nerve biopsies showing the presence of nonreplicative HCV-RNA in epineurial cells, in close spatial relationship with mononuclear inflammatory infiltrates, speaking in favor of HCV-mediated cellular inflammation [60, 61]. This is in keeping with the detection of positive-strand genomic HCV RNA in nerve and muscle tissue samples of patients with peripheral neuropathy, necrotizing arteritis and small-vessel lymphocytic vasculitis, findings suggestive of an immune-mediated pathogenesis, rather than a direct viral damage. Many patients develop a symmetrical sensory or sensorimotor axonal-type polyneuropathy, with sensory loss and weakness in distal regions of limbs, a pattern suggestive of a length-dependent process [62], similarly to the most frequent form of neuropathy encountered in HIV-1 infection [63]. Alternative common presentations include mononeuropathies and mononeuropathy multiplex, the latter producing a stocking-glove asymmetric neuropathy or overlapping syndrome (Figure 3). Cranial nerves are usually spared, although involvement of the abducens, facial, and motor trigeminal nerves has been reported. At variance with earlier reports, in more recent series of patients with HCV-associated neuropathy, sensory neuropathy represents the most prevalent form [64, 65]. The asymmetrical sensory variants include large-fiber sensory neuropathy (LFSN) and small-fiber sensory polyneuropathy (SFSN). LFSN is characterized by sensory loss, paresthesias, numbness, and cramps; conversely, SFSN, a painful condition targeting small myelinated and unmyelinated sensory axons, is clinically characterized by burning feet, tingling, restless leg syndrome, and, rarely, complex regional pain syndrome type 1 [66]; in some patients LFSN and SFSN may coexist. Intriguingly, patients with SFSN may disclose a pattern suggestive of ganglionopathy. Unusual forms of PNS involvement include pure motor polyneuropathies [67] and autonomic neuropathy [68]. 

The spectrum of peripheral neuropathies in HCV infection is not limited to axonal forms, but encompasses a number of demyelinating conditions. A patient with subacute sensory ataxia and IgMk cryoglobulin with demyelinating and axonal features was reported by Lippa et al. [69]. In addition, sensory demyelinating polyneuropathy, responsive to immunomodulatory treatment, has been also detected in subjects with polyclonal hypergammaglobulinemia or IgM monoclonal gammopathy, in the absence of CG [70], suggesting the occurrence of humoral immune-mediated demyelination. Other forms of HCV-related demyelinating conditions include the Lewis-Sumner syndrome [71] and chronic inflammatory demyelinating polyradiculoneuropathy [72]. 

## 5. Myopathies

The association between chronic HCV infection and myopathy is infrequent, and only few cases of noninflammatory and inflammatory myopathies have been reported so far. Clinical features of HCV-related myopathies widely range from progressive weakness to relapsing forms, and it is not unusual to find subjects with only mild elevation of muscle enzymes and/or moderate weakness, which leads to considerable diagnostic difficulties. 

In noninflammatory myopathies, pathological features are variegate and include vacuolar changes [73] or necrotizing myopathy [74], in association with slowly or progressive proximal weakness, and selective atrophy of type 2 fibers in relapsing myopathy. The occurrence of oxidative mitochondrial damage has been suggested in a patient with severe ptosis, diplopia, generalized weakness and respiratory involvement, complex III deficiency and ultrastructural alterations of mitochondrial shape and cristae [75]. Moreover, a pathogenic role for circulating cytokines and growth factors in mediating muscle damage has been advanced based on experimental findings showing that HCV promotes TNF-mediated apoptosis in myocytes [76]. 

At variance with noninflammatory myopathies, the clinical presentation of inflammatory forms is usually subacute and insidious, and muscle biopsies usually show various degrees of focal or diffuse muscle inflammation. Polymyositis is frequently reported in HCV infection, either without [77, 78] or with CG [79], being associated with interstitial lung disease in some patients. The presence of genomic HCV RNA, but not replicative intermediates in muscle specimens, a picture suggestive of an autoimmune HCV-triggered process, has been detected in patients with polymyositis [80, 81]. Additionally, evidence of complement activation with membrane attack complex deposition, in addition to cytotoxic T cell activation, has been obtained in a patient with inflammatory myopathy and muscle HCV RNA [82]. 

Dermatomyositis has been reported in a few patients with incidentally discovered HCV infection, as well as in subjects with long-term chronic HCV and hepatocellular carcinoma. Although the pathogenesis of HCV- or HCV-HCC-related dermatomyositis remains unexplained to date, a role for circulating anti-aminoacyl-tRNA synthetase antibodies, including anti-Jo1 and anti-Mi2 autoantibodies, has been suggested [83]. 

Alternative pathogenic mechanisms, involving HCV-induced oxidative DNA damage, have been advanced in patients with muscle deposition of HCV-RNA/HCV antigens and inclusion body myopathy, a muscular disorder whose classification under inflammatory forms is currently under debate [84, 85]. 

## Figures and Tables

**Table 1:** HCV-associated CNS and neuromuscular syndromes.

Disorders	Clinical Features
Neurological	
Stroke, TIA, lacunar syndromes	Focal signs
Acute encephalopathic forms	Confusion, altered consciousness, incontinence
Leukoencephalopathy	Multifocal signs and symptoms, cognitive dysfunction, tetraparesis, aphasia
Encephalomyelitis	Motor, sensory and sphincter deficits, seizures
Myelitis	Sensory ataxia, spastic paraplegia
Cognitive/Neuropsychiatric	
Fatigue	Sensation of physical and mental exhaustion
Psychiatric disorders	Depression, anxiety
Cognitive dysfunction	Alterations in verbal recall, working memory, sustained attention, concentration,
	learning skills
Peripheral Neuropathies	
Sensorimotor axonal polyneuropathies	Sensory loss, distal weakness
Large fibres sensory neuropathies	Reduced touch and proprioception sensations, sensory ataxia
Small fibres sensory neuropathies	Burning feet, pain, restless legs syndrome
Motor axonal polyneuropathies	Distal weakness
Mononeuropathies	Deep aching pain, truncular deficits
Mononeuropathy multiplex	Stocking-glove asymmetric neuropathy
Demyelinating forms	Sensory loss, distal weakness, areflexia
Myopathies	
Noninflammatory	Progressive proximal/generalized weakness, atrophy
Inflammatory	Progressive symmetrical proximal weakness, atrophy, dysphagia, interstitial lung disease
